# A short guide to histone deacetylases including recent progress on class II enzymes

**DOI:** 10.1038/s12276-020-0382-4

**Published:** 2020-02-19

**Authors:** Suk-Youl Park, Jeong-Sun Kim

**Affiliations:** 1 Pohang Accelerator Laboratory, Pohang University of Science and Technology, 80 Jigokro-127-Beongil, Nam-gu, Pohang, Gyeongbuk 37673 Republic of Korea; 2 Department of Chemistry, Chonnam National University, 77 Yongbong-ro, Buk-gu, Gwangju 61186 Republic of Korea

**Keywords:** Gene silencing, Protein folding

## Abstract

The interaction between histones and DNA is important for eukaryotic gene expression. A loose interaction caused, for example, by the neutralization of a positive charge on the histone surface by acetylation, induces a less compact chromatin structure, resulting in feasible accessibility of RNA polymerase and increased gene expression. In contrast, the formation of a tight chromatin structure due to the deacetylation of histone lysine residues on the surface by histone deacetylases enforces the interaction between the histones and DNA, which minimizes the chance of RNA polymerases contacting DNA, resulting in decreased gene expression. Therefore, the balance of the acetylation of histones mediated by histone acetylases (HATs) and histone deacetylases (HDACs) is an issue of transcription that has long been studied in relation to posttranslational modification. In this review, current knowledge of HDACs is briefly described with an emphasis on recent progress in research on HDACs, especially on class IIa HDACs.

## Cancer: Loosening DNA–Protein interactions

Targeting specific structural and functional features of enzymes involved in regulating the interactions between DNA and the histone proteins associated with it could lead to the development of more effective cancer therapeutics. Histone deacetylases (HDACs), enzymes which remove acetyl groups from histones, make the histones wrap more tightly around the DNA so that it becomes inaccessible to the initial steps in gene expression. Drugs that target these enzymes have shown limited efficacy due to lack of specificity and off-target toxicity. Jeong-Sun Kim at Chonnam National University, Gwangju, and Suk-Youl Park at Pohang Accelerator Laboratory, Pohang University of Science and Technology, South Korea, review the latest knowledge about class II HDACs. They suggest that their unique structural features and low enzymatic activity are important features to consider when designing new, more selective HDAC inhibitors.

## Introduction

**Fig. 1:**
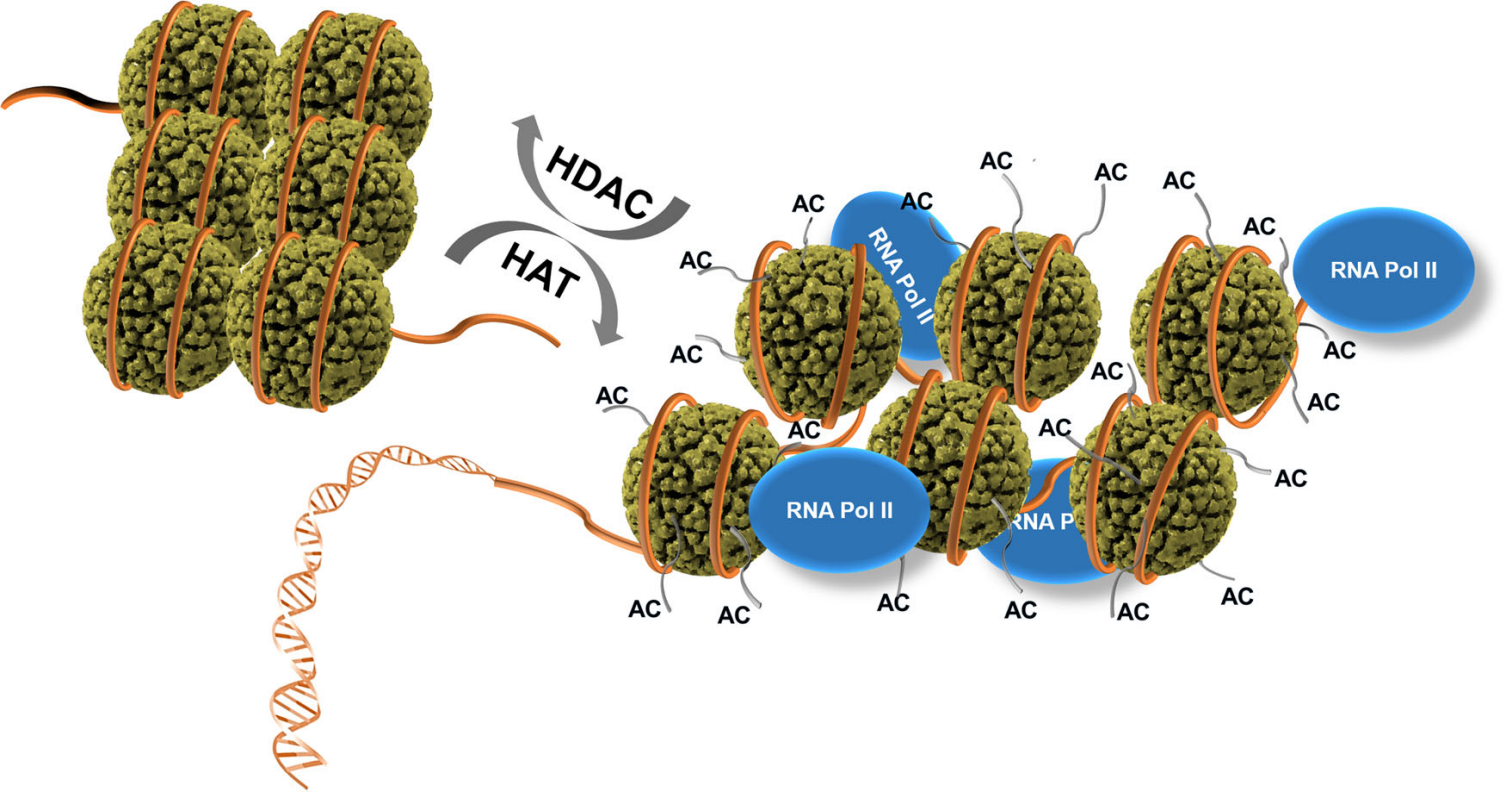
Regulation of gene expression and repression by histone acetylase (HAT) and histone deacetylase (HDAC). Acetylation (AC) of histone lysine residues by HAT, opening up the chromatin structure, allows binding of RNA polymerase II (RNA Pol II), while deacetylation of the histone lysine residues by HDAC leads to the closed chromatin conformation to be unable to bind RNA Pol II. Histones are displayed with dark green spheres. DNA wound around the histones is shown as an orange tube. The histone lysine residues are drawn with thin and short gray tails on histone spheres.

Long eukaryotic DNA is wrapped around histone proteins, leading to compact chromosomes. The compact nucleosome structure resulting mainly from the ionic interaction between the highly positively charged histones and the negatively charged DNA backbone restricts the access of the transcriptional machinery. The tight nucleosomes can become loose when the positive charge of the lysine residues on the histone surface is neutralized by acetylation performed by histone acetylase (HAT), which increases the accessibility of RNA polymerase II, resulting in gene expression. On the other hand, the recovery of a positive charge on the lysine side chain of the histone surface resulting from the action of histone deacetylase (HDAC) restores a compact chromatin structure, rendering access by RNA polymerase difficult, and thereby decreasing gene repression (Fig. 1). This mediation of gene expression by the acetylation and deacetylation of histones (a type of posttranslational modification) is a major gene expression regulation system in many eukaryotes, commonly referred to as the epigenetic control of eukaryotic gene transcription. The disruption of the balance between HAT and HDAC activities can result in the aberrant expression of a specific gene that ultimately leads to the instability of chromatic structure and epigenetic diseases^1,2^. Hence, the precise control of the activities of HATs and HDACs is important for the exact and timely expression of various genes associated with signal transduction and cell growth and death^2^. An imbalance between HAT and HDAC activities can also be caused by the repression of the intrinsic enzyme activity of HAT or HDAC. When HAT activity is inhibited, the timely expression of a target gene is hindered. On the other hand, the inhibition of HDAC activity keeps a continuous expression of a target gene. In this context, the control of HDAC activity by HDAC inhibitors has been targeted for the development of anticancer strategies as well as therapies for human diseases derived from cardiovascular, metabolic, and neurodegenerative disorders^3–8^.

Eighteen human HDACs are grouped into four classes based on their primary homology to yeast HDACs. Among these groups, class I and II HDACs play a major role in the lysine deacetylation of N-terminal histone tails. HDACs interact with several partners through distinct domains. Both class I HDAC3 and IIa HDACs interact with two closely related corepressors: silencing mediator for retinoid and thyroid receptors (SMRT) and nuclear receptor corepressor (N-CoR). SMRT/N-CoR is associated with the sequence-specific DNA-binding domain of BCL6, which is involved in B-cell activation and differentiation, inflammation, and cell-cycle regulation^9–11^. Interestingly, HDAC3 and class IIa HDACs are catalytically inactive in their solitary state. However, when HDAC3 is bound to SMRT/N-CoR, it becomes enzymatically active regardless of the presence a class IIa HDAC^12,13^. In contrast, class IIa HDACs do not show any significant enhancement of lysine deacetylase activity after binding to the SMRT/N-CoR proteins. The SMRT/N-CoR corepressors provide a structural link between active HDAC3 and inactive class IIa HDACs. Therefore, the role of class IIa HDACs and the biological relevance of these observations remain unclear.

## Chromatin remodeling via histone modification

The human genome consists of a set of DNA compacted within 23 chromosome pairs containing approximately 6,469.66 megabase pairs, which encode over 20,000 genes. If the DNA from a single human cell was to be stretched out, it would be ~2 m long. The average human cell diameter is ~100 µm, and the nucleus is ~30 µm. However, the total DNA in each cell fits into a space with a diameter of only 6 microns, because DNA exists in chromatin form in the nucleus. Chromatin forms a “beads-on-a-string” structure referred to as a nucleosome. The “beads” are octamer histone proteins, and the “string” is double-stranded DNA molecule. Approximately 147 base pairs wrap around each 8-component histone protein to form a nucleosome of 11 nm in diameter. The “beads-on-a-string” structure forms a more condensed solenoid coil conformation with six nucleosomes constituting the 30 nm chromatin fibers of packed nucleosomes. Then, the supercoiled chromatin fibers form a chromosome. The details are provided in a review by Donald E. Olins & Ada L. Olins^14^. This chromatin conformation must be controlled to allow access to DNA and RNA polymerase for replication and transcription. Basically, histone tails are alkaline and associate with DNA, where the positive surface charges on their lysine and arginine residues contribute to binding with the negatively charged DNA phosphate component. To date, five types of histones have been identified: H1 (homologous H5), H2A, H2B, H3, and H4. H1 and H5 are linker histones for the solenoid chromatin structure. H2A, H2B, H3, and H4 are the core proteins that associate with DNA, forming the thread-like structure. Histones can be modified by the acetylation or deacetylation of lysine residues by HATs or HDACs to regulate the interaction between histones and DNA. The acetylated histone modification affects the nucleosome space for RNA polymerase sliding to achieve specific gene expression. In contrast, histone deacetylation represses gene expression by inducing a closed chromosomal conformation. Thus, the gene can be turned on or off depending on the modifications to the histone proteins determining DNA exposure, which is referred to as epigenetic regulation.

## HDAC classes

**Fig. 2:**
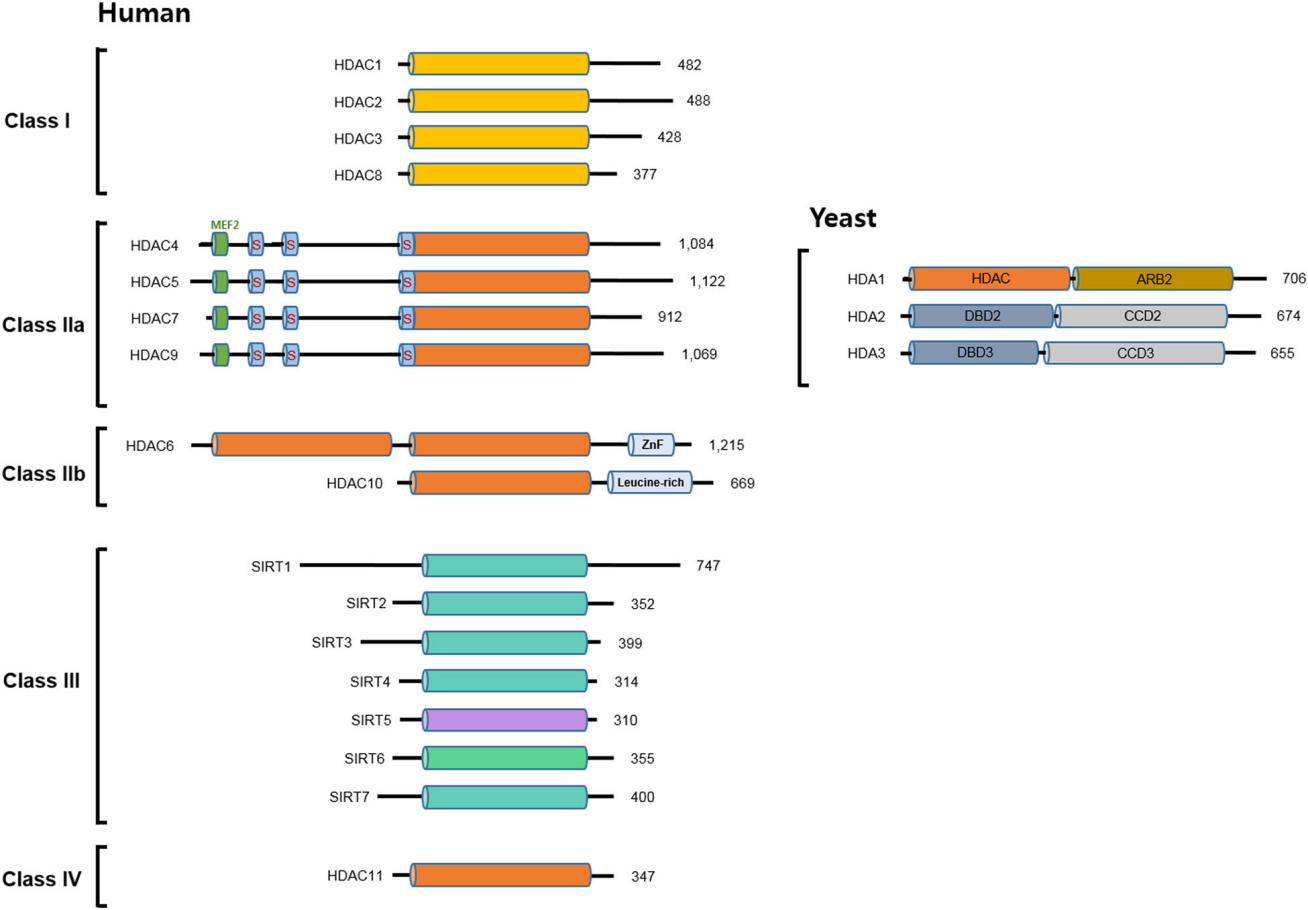
Classification and domain composition of human and yeast HDACs. The total number of amino acid residues in each HDAC is shown on the right of each protein. For simplicity, the longest isoforms are shown. Catalytic domains of HDACs are shown in cylinders. Myocyte enhancer factor-2 (MEF2)-binding motifs are depicted as short green cylinders, whereas 14-3-3 binding motifs are shown as short blue cylinders labeled with “S” (for serine). ZnF, ubiquitin-binding zinc finger.

HDACs can be divided into two families based on the presence of a conserved deacetylase domain and their dependence on specific cofactors: the histone deacetylase family and the sirtuin protein family. The deacetylase family is subdivided into class I (HDAC1, 2, 3, and 8), class II (HDAC4, 5, 6, 7, 9, and 10), and class IV (HDAC11) based on sequence similarity to yeast deacetylases, which are zinc-dependent amidohydrolases. The class II enzymes are further divided into class IIa and class IIb, according to their domain compositions. The sirtuin proteins are classified within the class III HDACs, which require nicotinamide adenine dinucleotide (NAD) as a cofactor for their catalytic function. To date, 18 mammalian HDACs have been reported and grouped into the above different classes (Fig. 2).

Class I HDACs are highly homologous to the yeast HDAC Rpd3 and are composed of an entirely conserved deacetylase domain compared with other classes. They are ubiquitously expressed and predominantly located in the nucleus, where they present strong deacetylase activity toward histones. They also act as catalytic subunits to repress targeted genes in complex with their cognate corepressors, which is regulated by inositol phosphates^15^. In recent studies, class I HDACs have been shown to deacetylate nonhistone proteins such as AMP-activated protein kinase and cohesion subunit SMC3 as well as transcription regulators to control their activity^16^. HDAC1 and 2 form a complex with the nucleosome remodeling and deacetylase complex (NuRD), transcriptional regulatory protein Sin3A, corepressor of REST (CoREST), and the mitotic deacetylase complex (MiDAC), while HDAC3 is recruited to the SMRT/N-CoR corepressor complex (reviewed by Ayer)^17,18^. However, HDAC8 functions alone without forming a large complex^19^. Mihaylova and Shaw^20^ and Seto and Yoshida^21^ provided an overview of the various HDAC class I complexes along with a detailed discussion.

Class II HDACs share high homology with yeast HDA1 and exhibit a conserved deacetylase domain at their C-terminus. The subdivided class IIa HDACs (HDAC4, 5, 7, and 9) harbor a unique adapter domain in the N-terminus that forms a binding site for the DNA-binding transcription factor MEF2, and the subsequent 3 ~ 4 phosphorylation sites act as regulatory signals for the association of 14-3-3 proteins, which can shuttle between the cytoplasm and nucleus in response to various regulatory signals^22^. Class IIb HDACs (HDAC6 and 10) exhibit a characteristic long extra extension at the C-terminus, known as a tail domain. These two class IIb HDACs also differ; while HDAC6 contains two deacetylase domains and a C-terminal zinc finger ubiquitin-binding domain, HDAC10 has only one deacetylase domain and a leucine-rich repeat domain at its C-terminus^23–27^. These enzymes are typically found in the cytoplasm. Class IIa HDACs also form a large complex with the SMRT/N-CoR-HDAC3 complex^28^. Intrinsically, class IIa HDACs present very low enzymatic activity. In the case of HDAC4, the H976Y (histidine to tyrosine) HDAC4 mutant shows a 1000-fold increase in deacetylase activity compared with the wild-type. The tyrosine residue in the catalytic site is conserved in class I HDACs, whereas tyrosine is substituted for histidine in class IIa HDACs. Thus, class IIa HDACs might play a role as a deacetylase involving low enzymatic activity or could present specific targets that remain undiscovered. Class IIb HDACs are poorly understood. HDAC6 is involved in the deacetylation of α-tubulin, cortactin, chaperones, and IFNαR, and is implicated in the regulation of autophagy as well as hepatic metabolism^29^. Mihaylova and Shaw^20^ and Seto and Yoshida^21^ provided an overview of the class II HDACs.

HDAC class IV includes only HDAC11, which is homologous to yeast Hos3 and shares a catalytic domain with class I and class II HDACs^30^. It is related to the DNA replication factor CDT1 and interleukin 10 expression^31^. HDAC11 has also not been well studied.

The class III HDACs are homologous to the yeast silent information regulator 2 (Sir2) required for transcription silencing and are widely conserved proteins in many organisms from bacteria to humans^32^. The deoxyhypusine synthase-like NAD/FAD-binding domain of class III HDACs is a distinct characteristic among HDACs. Seven Sir2-like proteins (SIRT1-7) have been reported in humans, which are referred to as sirtuins^33^. A unique characteristic of these sirtuins is that they exhibit other enzymatic activities, such as momo-ADP-ribosyltransferase activity. SIRT1 possesses the most robust histone deacetylase activity. Interestingly, SIRT5 shows lysine desuccinylase and demalonylase activity^34^. These proteins may be found in the nucleus (SIRT1, SIRT2, SIRT3, SIRT6, and SIRT7), cytoplasm (SIRT1 and SIRT2), or mitochondria (SIRT3, SIRT4, and SIRT5). SIRT5 has been well investigated. The review by Seto and Yoshida^21^ provides more detail on this topic.

## HDAC structures and catalytic mechanisms

To date, 248 HDAC structures have been reported in the Protein Data Bank. Among these structures, ~100 structures are for human HDACs (HDAC8, 47; HDAC4, 17; HDAC6, 17; HDAC2, 6; HDAC7, 5; Sirtuin-3, 4; Sirtuin-5, 4). The first solved HDAC structure was for a bacterial histone deacetylase-like protein (HDLP) from *Aquifex aeolicus* that exhibits the α/β deacetylase fold of a previously determined arginase. This arginase is a metalloenzyme that uses a manganese ion as a catalytic cofactor, suggesting that the HDACs have evolved from a common metalloenzyme ancestor^35^. The details are provided in Lombardi et al.’s review^36^.

Class I, II, and IV HDACs are zinc-dependent. Initially, the positioning of the zinc ion is coordinated by one histidine, two aspartates, and two water molecules at the active site. As a substrate, the acetamide carbonyl group of the acetylated lysine replaces one water molecule and interacts with the hydroxyl group of a tyrosine residue via hydrogen bonding. The tyrosine residue may undergo a conformational transition from an “out” conformation to an “in” conformation to accommodate substrate binding. Then, the other water molecule is deprotonated by a nearby histidine nucleophile through the His–Asp charge-relay network, which is a typical system for the polarization and activation of a water molecule as a nucleophile. There are two tandemly located histidine residues near the active site. The first histidine has been suggested to act as a general electrostatic catalyst to activate the water molecule, while the second histidine functions to accept a proton as a general base^37^. After the oxygen of the deprotonated water attacks the lysine carbonyl group, oxyanions and tetrahedral intermediates are generated. Finally, when the second histidine serves as the proton-to-amine moiety of the intermediate, acetate and lysine are generated. The details of this process are provided are in Lombardi et al.’s review^36^. In class IIa, the tyrosine residue is replaced with a histidine, which seems to be important for the formation of the tetrahedral intermediate. Thus, the enzymatic activity of the members of class IIa is relatively low compared with that of other classes.

The class III HDACs are NAD-dependent enzymes. The reported class III structures exhibit a catalytic cleft between a large Rossmann fold domain and a small zinc-binding domain^38^. The cleft residues are conserved in sirtuin family proteins and form a tunnel for NAD^+^ and acetylated lysines or large substrates, such as succinated lysines and malonated lysines^34^. The catalytic reaction begins with the cleavage of nicotine amide via the nucleophilic addition of acetamide oxygen of the acetylated lysine, which generates a C1′-O-alkylamidate intermediate. The histidine residue of the active site activates the 2′-hydroxyl group of the first intermediate by deprotonation. The activated oxygen attacks the C1′-O-alkylamidate to form the 1′,2′-cyclic tetrahedral intermediate. Then, after the tetrahedral carbon intermediate is attacked by a water molecule, deacetylated lysine and 2′-O-acetyl-ADP ribose are generated. The 2′-O-acetyl-ADP ribose can be spontaneously converted into 3′-O-acetyl-ADP ribose. The details are provided in Seto and Yoshida’s review^21^.

## Class I and II HDACs from yeast

Among the yeast HDACs, class I Rpd3 and class II HDA1 play major roles in the transcriptional repression of gene expression^39–42^. Class I Rpd3p associates with several subunits (e.g., scSin3p, scSap30p, scSde3p, and scPho23p) to form a multiprotein Rpd3 complex, whereas dimeric HDA1 assembles with two noncatalytic subunits, HDA2 and HDA3, to form a heterotetrameric HDA1 complex that specifically deacetylates the H2B and H3 subunits of histones^43–45^. These two complexes are recruited to specific promoter loci through sequence-specific DNA-binding proteins for transcriptional repression. Subsequently, adjacent nucleosomes are precisely modified at the lysine residues of histones. They also deacetylate the N-terminal tails of the histones via a nonspecific DNA-binding mechanism^42,46^. The HDA1 complex is related to human class II HDACs. The dimeric HDA1 of the HDA1 complex only presents deacetylase catalytic activity. HDA2 and HDA3 are also essential for deacetylase activity in vivo and in vitro^41,47^. HDA1 is associated with specific promoter sites in yeast, while the HDA2–HDA3 subcomplex binds to nonspecific DNA sites^41,48^. HDA1 comprises an N-terminal deacetylase domain and a C-terminal argonaute-binding protein 2 (ARB2), which is essential for histone methylation, heterochromatin assembly, and siRNA generation^49^. The recently identified ARB2 domain mediates homodimer formation and then enables the binding of histones, H2A–H2B dimers or H3–H4 tetramer^50^. Both HDA2 and HDA3 present an N-terminal DNA-binding domain (DBD) and a C-terminal coiled–coil domain (CCD). The CCDs are utilized for the HDA2–HDA3 heterodimeric subcomplex. The DBD domains show structural homology to the C-terminal helicase lobes of the SWI2/SNF2 chromatin-remodeling domains of the Rad54 family enzymes^48^. The HDA2–HDA3 subcomplex was suggested to act as a DNA-binding scaffold protein for an enzymatically competent yeast class II HDA1 HDAC complex^48^. HDA2 and HDA3 homologues have not been identified in humans. According to extensive structural and functional studies, the Huber group^48^ proposed that both N-terminal DBDs of the two structural subunits of the HDA2–HDA3 complex serve as unspecific DNA-binding modules and as anchors to position HDA1 in the proximity of the H2B and H3 histone tails.

**Fig. 3:**
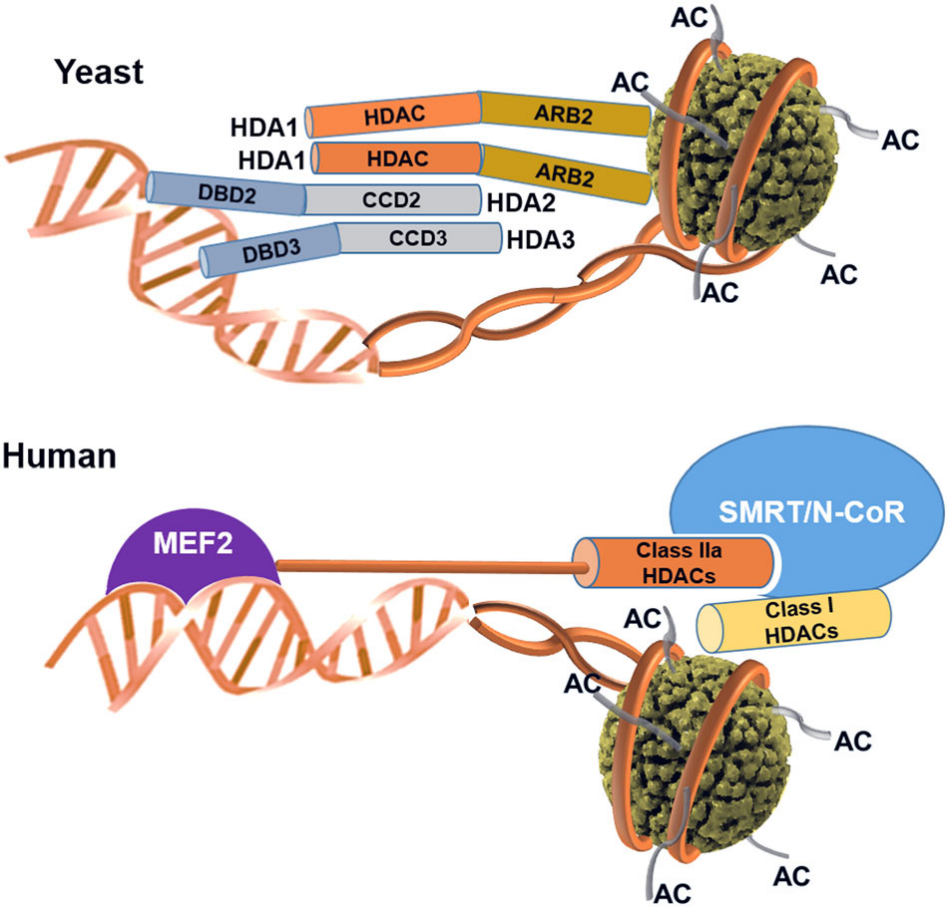
Proposed model of class II yeast and human HDAC complex. In yeast, the HDA1 complex may form a larger complex including interactions of the homodimeric HDA1 ARB2 domain with histones, the heterodimeric HDA2–HDA3 DBDs with nonspecific DNA, and the N-terminal catalytic domain of HDA1 with the HDA2-HDA3 CCDs. In human, class IIa HDACs may form a bridge between the SMRT–HDAC3 complex and MEF2 transcription factors rather than the enzymatic function of histone deacetylase.

On the basis of the analysis of the domain organization and interactions of the homodimeric HDA1 ARB2 domains with histones, the heterodimeric HDA2–HDA3 DBDs with nonspecific DNA, and the N-terminal deacetylase domain of HDA1 with the HDA2–HDA3 CCDs, the whole HDA1 complex may form a larger complex including histones and DNA for the global deacetylation of nucleosomes^48,50^ (Fig. 3).

## Unique characteristics of class IIa HDACs

**Fig. 4:**
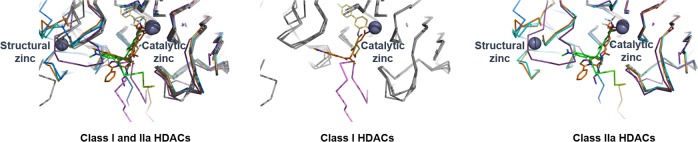
Structural comparison of class I and class IIa HDAC catalytic domains with inhibitors and bound peptides. Superposed class I HDACs (gray) and class IIa (cyan, orange, blue, and magenta) were drawn as a ribbon model, and the catalytic and structural zinc ions (black) are displayed with balls. The bound inhibitors and peptides that were displayed with stick models were differentiated with colors.

As mentioned above, class II HDACs exhibit an extended region at the N-terminus and/or C-terminus. Interestingly, class IIa HDACs present a nuclear localization sequence in the N-terminal region; therefore, they are commonly found both in the nucleus and in the cytoplasm. Along with the attached regions, the catalytic domains of class II HDACs exhibit another zinc ion-binding site referred to as the structural zinc ion-binding subdomain. Many of the class IIa-specific residues are found in this structural zinc ion-binding site and at the entry site to the catalytic site (Fig. 4). The catalytic activity of class II HDACs is extremely low (~1/1000-fold) compared with that of class I HDACs, which is attributed to the catalytic Tyr residue being replaced with a His residue.

Indeed, HDAC4, a member of class IIa HDACs, gained its deacetylase activity when this Tyr was substituted for the His residue. Therefore, it is not clear which roles can be conferred to class II HDACs, even though clear biological phenomena are observed when each of the class II HDACs are ablated.

## HDAC-related diseases and inhibitors

Imbalanced HAT and HDAC activities can affect the compaction level of a local chromatin region and result in improper expression of specific genes, ultimately leading to genomic instability and epigenetic diseases^1,2^. Therefore, the precise control of HATs and HDACs is required for the regulated expression of various genes associated with signal transduction, cell growth, and cell death^3^. Anti-histone deacetylase drugs such as SAHA (Vorinostat, Zolinza™, Merck & Co, Inc., USA) and FK228 (Romidepsin, Istodax™, Celgene Corp., USA) have been used for the treatment of cutaneous T-cell lymphoma (CTCL). Most recently, belinostat (PXD101, BELEODAQ™, Spectrum Pharmaceuticals, Inc.) was approved by the FDA for use against peripheral T-cell lymphoma (PTCL) in 2014, and panobinostat (Farydak, Novartis Pharmaceuticals) was licensed for the treatment of multiple myeloma in 2015^51^. Over the past decade, due to dose-limiting toxicities and nonselectivity, a high level of interest has been focused on a variety of combination therapies involving other anticancer agents and their translation into preclinical and clinical studies. These combinational therapies include DNA repair pathway drugs, radiotherapy, topoisomerase inhibitors, epigenetic modifiers, and immune checkpoint inhibitors, and have shown significant synergistic efficacy. Thus, HDAC inhibitors still seem to be a promising group of anticancer drugs, even though many biological effects of HDAC inhibitors remain unclear. However, one of the major shortcomings of these HDAC inhibitors is limited therapeutic efficacy against solid tumors as single therapeutic agents, and resistance to these inhibitors remains largely unknown. The details are reviewed by Eckschlager et al.^52^.

## Promising HDAC small-molecule inhibitors and their limitations

Targeting the catalytic activity of an HDAC has been the predominant method of generating HDAC inhibitors. Accordingly, a large number of competitive HDAC inhibitors directed against the catalytic pocket have been developed and tested in various preclinical and clinical stages. Among these inhibitors, SAHA (vorinostat) and FK288 (romidepsin) were the first FDA-approved HDAC inhibitors for use in anticancer clinical fields. Valporic acid and CI994 are undergoing phase III clinical trials for use against cervical and ovarian cancer, myeloma, and lung cancer^53^. Several other inhibitors, such as mocentinostat, PCI24781, and MS275-SNDX-275, are also being tested in phase I/II clinical trials against solid and hematological cancers. However, similar to other anticancer therapeutics, HDAC inhibitors have shown only limited efficacy, along with dose-limiting toxicity that is associated with side effects. These side effects include thrombocytopenia, neutropenia, nausea, vomiting, diarrhea, and fatigue. The most troubling effect is cardiac toxicity, including ventricular arrhythmia. Indeed, prior to FDA approval, there were six deaths in patients treated with romidepsin^54^. In fact, one of the common problems associated with the currently available HDAC inhibitors is their low degree of target HDAC selectivity against other classes of HDACs as well as closely related members of the same class, such as HDAC4, HDAC5, HDAC7, and HDAC9, with different tumor-suppressor functions. Therefore, the improvement of HDAC isoform specificity is likely one of the most pressing concerns to be addressed to minimize off-target toxicity.

The generation of metal-chelating inhibitors with the aim of inhibiting the catalytic activity of HDACs is a widely used approach, partly because it is relatively straightforward to design small chelating molecules that block the metal-dependent hydrolysis of an acetylated substrate. However, owing to the highly conserved nature of metals, especially zinc-binding sites, a large fraction of metalloprotein inhibitors present the possibility of cross-reactivity with various other metalloenzymes such as arginase and HDAC-related deacetylases, which are histone deacetylase-like proteins^55^. Therefore, for a given HDAC, a better understanding of the exact nature of the zinc-binding site and its nearby interaction pockets could be crucial for overcoming the lack of specificity associated with non-metal-chelating inhibitors or achieving synergetic effects with the metal-chelating moiety.

## The N-terminal domain as an alternative drug target for class IIa HDACs

The N-terminal domain (NTD) of the class IIa HDACs is a functionally unique adaptor domain that binds transcription factors and mediates regulatory signals, which distinguishes these enzymes from other HDACs. The class IIa HDAC NTD contains interaction sites for members of the myocyte enhancer factor-2 (MEF2) family of transcription factors and conserved serine residues that undergo signal‐dependent phosphorylation, which leads to the nuclear export of the enzymes and the de‐repression of their targets (Fig. 2). However, much evidence suggests that other transcription factors are class IIa HDAC partners, such as Runx2, calmodulin‐binding transcription activator, and serum response factor^56–58^. Under basal conditions, class IIa HDACs are unphosphorylated and are located in the nucleus, where they are recruited to their target genes through interaction with transcription factors, enabling their transcription-repressive function. This suggests that the class IIa HDAC NTD may serve as an alternative target for the development of class IIa anti-HDAC therapeutics. However, it should be noted that class IIa HDACs are phosphorylated in response to specific signals, leading to the disruption of the interaction with transcription factors, their export to the cytoplasmic compartment, and the de‐repression of their targets. Previous reviews have provided detailed descriptions of the extracellular signals and kinases involved in the phosphorylation of class IIa HDACs. Therefore, the inhibition of class IIa HDAC NTD function represents a target-restricted strategy that is designed to antagonize a subset of class IIa HDAC functions that are dysregulated in cancer.

## Advantages of targeting class IIa HDACs

Intriguingly, the other unique characteristic of the class IIa HDACs is their lack of measurable enzymatic activity. Although they harbor a highly conserved catalytic domain, they exhibit minimal deacetylase activity against acetylated histones. To date, no histone or other protein substrates have been identified for class IIa HDACs. In fact, their enzymatic activity depends on their recruitment into a multiprotein complex containing HDAC3 and SMRT/N-CoR^12,59^. It has been proposed that class IIa HDACs may not actually act as enzymes in histone deacetylation and that they instead act as adaptors of repressor complexes^12^. The class IIa HDACs appear to be expressed in a tissue‐specific manner, and have been shown to exert their transcription-repressive function in cardiac and vascular hypertrophy, myoblast differentiation, neuronal cell survival, and neurodegenerative disorders^60^. Therefore, class IIa HDACs are currently considered critical regulators of specific developmental and differentiation processes^61^. However, most of the studies demonstrating their tissue specificity have employed only in vitro experimental approaches. The details are described in review by Verdin et al.^57^. NTD inhibitors targeting class IIa HDACs have been poorly studied. This line of research is expected to reduce the likelihood of undesired cross-reactivity of inhibitors against other classes of HDACs.

**Fig. 5:**
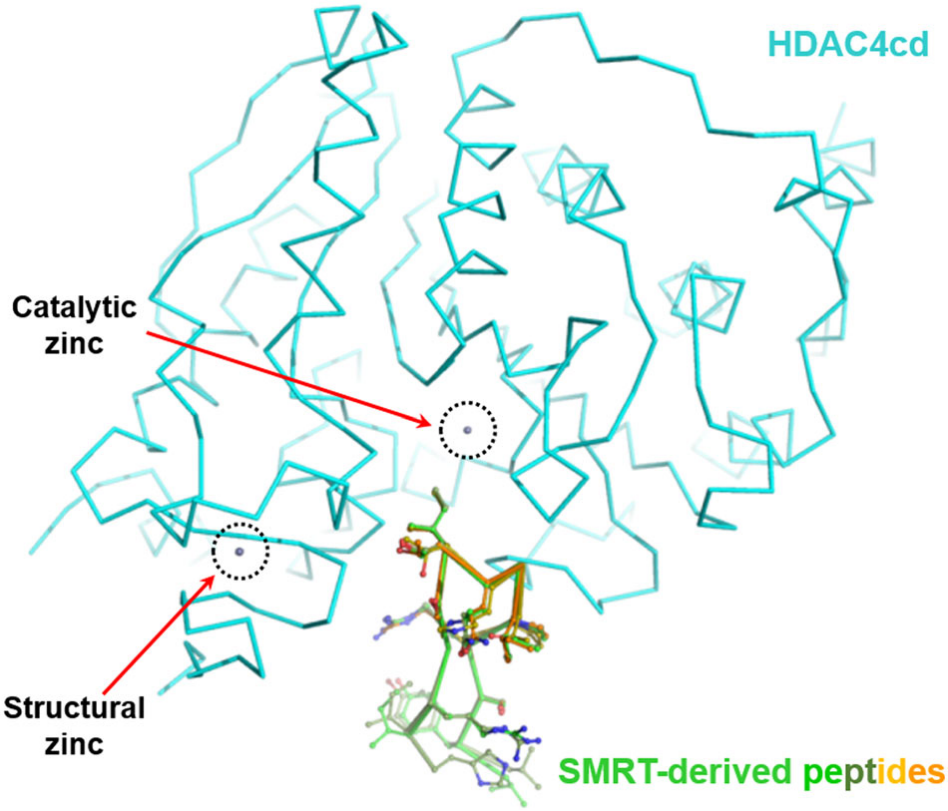
Complex structure of the class IIa HDAC4 catalytic domain (HDAC4cd) and SMRT-derived peptides (SPs). The bound SPs are superposed. HDAC4cd and the bound peptides are differentiated by alternating colors. Side chains of the peptides are displayed with ball-and-stick models. The catalytic and structural zinc ions are drawn with balls and indicated with black-dotted circles.

**Fig. 6:**
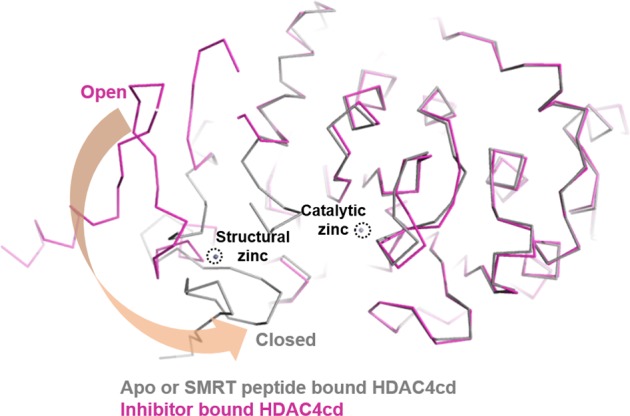
Open and closed conformation of the class IIa HDAC4 catalytic domain (HDAC4cd). Two alternative conformations of the structural zinc ion-binding site. The open conformation (magenta) and closed conformation (gray) are differentiated. The catalytic and structural zinc ions are drawn with balls and indicated with black-dotted circles.

Another potential advantage of targeting the class IIa HDACs is the availability of a peptido-mimetic approach for the design of class IIa-specific inhibitors based on complex structures with SMRT peptides^62^. Structural and biochemical studies have shown that the entrance of the HDAC4 catalytic site interacts with SMRT glycine–serine–isoleucine motif-containing peptides (Fig. 5). As described above, class IIa HDACs exhibit a catalytic zinc-containing deacetylase domain and a flexible zinc-binding subdomain^63,64^. According to the reported structural analyses of the inhibitor-bound HDAC4 catalytic domain, the structural zinc ion-binding region can adopt two distinct conformations, either “closed” or “open” (Fig. 6). The apo-structure forms the “closed” conformation, which may provide a substrate path leading to the catalytic zinc ion at the active site, while inhibitor-bound structures show the “open” conformation, with a disordered structural zinc ion-binding subdomain^63,65^. Thus, the closed conformation has been speculated to be a biologically relevant structure that does not interfere with the complex with SMRT-HDAC3^62^. Interestingly, the structures of HDAC4 complexed with SMRT-derived peptides (SPs) exhibit a closed conformation. Therefore, an SP-based inhibitor may provide a better rationale for the design of biologically active HDAC4 inhibitors, which may provide high specificity for class IIa HDACs compared with other known chemical inhibitors.

## New biological approaches related to class IIa HDACs and concluding remarks

As mentioned above, class IIa HDACs exhibit low deacetylase activity, and their domains form a large complex with the SMRT/N-CoR-HDAC3 complex for the repression of specific genes. Studies of class IIa HDACs have reported that the deacetylase activity of HDAC4 is not increased when it forms a complex with SMRT^28^. Our recent work also showed that the catalytic entry site of HDAC4 is occupied by SMRT-binding motif residues, based on the crystal structure of the HDAC4 catalytic domain and SMRT glycine–serine–isoleucine motif complex^62^. This evidence strongly suggests that class IIa HDACs function as bridge molecules between the SMRT-HDAC3 complex and transcription factors rather than acting as deacetylase enzymes for acetylated histones (Fig. 3). The identification of real substrates of class IIa HDACs independent of the SMRT-HDAC3 complex is still necessary to evaluate their physiological roles considering their intrinsic low enzymatic activity. In addition, studies of the association and inhibition between HDAC4/5/7 and SMRT/N-CoR are needed to determine how they change the acetylation pattern of target genes. These approaches will answer fundamental questions about class IIa HDACs.

## References

[1] Di Gennaro E, Bruzzese F, Caraglia M, Abruzzese A, Budillon A (2004). Acetylation of proteins as novel target for antitumor therapy: review article. Amino Acids.

[2] Vahid F, Zand H, Nosrat-Mirshekarlou E, Najafi R, Hekmatdoost A (2015). The role dietary of bioactive compounds on the regulation of histone acetylases and deacetylases: a review. Gene.

[3] Eom GH, Kook H (2015). Role of histone deacetylase 2 and its posttranslational modifications in cardiac hypertrophy. BMB Rep..

[4] Kalin JH (2018). Targeting the CoREST complex with dual histone deacetylase and demethylase inhibitors. Nat. Commun..

[5] Kazantsev AG, Thompson LM (2008). Therapeutic application of histone deacetylase inhibitors for central nervous system disorders. Nat. Rev. Drug Discov..

[6] Liu Kwei-Yan, Wang Li-Ting, Hsu Shih-Hsien (2018). Modification of Epigenetic Histone Acetylation in Hepatocellular Carcinoma. Cancers.

[7] Minucci S, Pelicci PG (2006). Histone deacetylase inhibitors and the promise of epigenetic (and more) treatments for cancer. Nat. Rev. Cancer.

[8] Yao R (2018). Scriptaid inhibits cell survival, cell cycle, and promotes apoptosis in multiple myeloma via epigenetic regulation of p21. Exp. Hematol..

[9] Kerckaert JP (1993). LAZ3, a novel zinc-finger encoding gene, is disrupted by recurring chromosome 3q27 translocations in human lymphomas. Nat. Genet..

[10] Ye BH (1993). Alterations of a zinc finger-encoding gene, BCL-6, in diffuse large-cell lymphoma. Science.

[11] Shaffer AL (2000). BCL-6 represses genes that function in lymphocyte differentiation, inflammation, and cell cycle control. Immunity.

[12] Fischle W (2002). Enzymatic activity associated with class II HDACs is dependent on a multiprotein complex containing HDAC3 and SMRT/N-CoR. Mol. Cell.

[13] Guenther MG, Barak O, Lazar MA (2001). The SMRT and N-CoR corepressors are activating cofactors for histone deacetylase 3. Mol. Cell Biol..

[14] Olins DE, Olins AL (2003). Chromatin history: our view from the bridge. Nat. Rev. Mol. Cell Biol..

[15] Watson PJ, Fairall L, Santos GM, Schwabe JW (2012). Structure of HDAC3 bound to co-repressor and inositol tetraphosphate. Nature.

[16] Xiong B, Lu S, Gerton JL (2010). Hos1 is a lysine deacetylase for the Smc3 subunit of cohesin. Curr. Biol..

[17] Ayer DE (1999). Histone deacetylases: transcriptional repression with SINers and NuRDs. Trends Cell Biol..

[18] Wen YD (2000). The histone deacetylase-3 complex contains nuclear receptor corepressors. Proc. Natl Acad. Sci. USA.

[19] Hu E (2000). Cloning and characterization of a novel human class I histone deacetylase that functions as a transcription repressor. J. Biol. Chem..

[20] Mihaylova MM, Shaw RJ (2013). Metabolic reprogramming by class I and II histone deacetylases. Trends Endocrinol. Metab..

[21] Seto E, Yoshida M (2014). Erasers of histone acetylation: the histone deacetylase enzymes. Cold Spring Harb. Perspect. Biol..

[22] Muslin AJ, Xing H (2000). 14-3-3 proteins: regulation of subcellular localization by molecular interference. Cell Signal.

[23] Grozinger CM, Schreiber SL (2000). Regulation of histone deacetylase 4 and 5 and transcriptional activity by 14-3-3-dependent cellular localization. Proc. Natl Acad. Sci. USA.

[24] Wang AH (2000). Regulation of histone deacetylase 4 by binding of 14-3-3 proteins. Mol. Cell Biol..

[25] McKinsey TA, Zhang CL, Olson EN (2000). Activation of the myocyte enhancer factor-2 transcription factor by calcium/calmodulin-dependent protein kinase-stimulated binding of 14-3-3 to histone deacetylase 5. Proc. Natl Acad. Sci. USA.

[26] Kao HY, Downes M, Ordentlich P, Evans RM (2000). Isolation of a novel histone deacetylase reveals that class I and class II deacetylases promote SMRT-mediated repression. Genes Dev..

[27] Zhang H, Okada S, Hatano M, Okabe S, Tokuhisa T (2001). A new functional domain of Bcl6 family that recruits histone deacetylases. Biochim. Biophys. Acta.

[28] Hudson GM, Watson PJ, Fairall L, Jamieson AG, Schwabe JW (2015). Insights into the recruitment of class IIa histone deacetylases (HDACs) to the SMRT/NCoR transcriptional repression complex. J. Biol. Chem..

[29] Winkler R (2012). Histone deacetylase 6 (HDAC6) is an essential modifier of glucocorticoid-induced hepatic gluconeogenesis. Diabetes.

[30] Gao L, Cueto MA, Asselbergs F, Atadja P (2002). Cloning and functional characterization of HDAC11, a novel member of the human histone deacetylase family. J. Biol. Chem..

[31] Glozak MA, Seto E (2009). Acetylation/deacetylation modulates the stability of DNA replication licensing factor Cdt1. J. Biol. Chem..

[32] Brachmann CB (1995). The SIR2 gene family, conserved from bacteria to humans, functions in silencing, cell cycle progression, and chromosome stability. Genes Dev..

[33] Frye RA (1999). Characterization of five human cDNAs with homology to the yeast SIR2 gene: Sir2-like proteins (sirtuins) metabolize NAD and may have protein ADP-ribosyltransferase activity. Biochem. Biophys. Res. Commun..

[34] Du J (2011). Sirt5 is a NAD-dependent protein lysine demalonylase and desuccinylase. Science.

[35] Hai Y, Shinsky SA, Porter NJ, Christianson DW (2017). Histone deacetylase 10 structure and molecular function as a polyamine deacetylase. Nat. Commun..

[36] Lombardi PM, Cole KE, Dowling DP, Christianson DW (2011). Structure, mechanism, and inhibition of histone deacetylases and related metalloenzymes. Curr. Opin. Struct. Biol..

[37] Wu R, Lu Z, Cao Z, Zhang Y (2011). Zinc chelation with hydroxamate in histone deacetylases modulated by water access to the linker binding channel. J. Am. Chem. Soc..

[38] Finnin MS, Donigian JR, Pavletich NP (2001). Structure of the histone deacetylase SIRT2. Nat. Struct. Biol..

[39] Rundlett SE, Carmen AA, Suka N, Turner BM, Grunstein M (1998). Transcriptional repression by UME6 involves deacetylation of lysine 5 of histone H4 by RPD3. Nature.

[40] Vogelauer M, Wu J, Suka N, Grunstein M (2000). Global histone acetylation and deacetylation in yeast. Nature.

[41] Wu J, Carmen AA, Kobayashi R, Suka N, Grunstein M (2001). HDA2 and HDA3 are related proteins that interact with and are essential for the activity of the yeast histone deacetylase HDA1. Proc. Natl Acad. Sci. USA.

[42] Kurdistani SK, Robyr D, Tavazoie S, Grunstein M (2002). Genome-wide binding map of the histone deacetylase Rpd3 in yeast. Nat. Genet.

[43] Kasten MM, Dorland S, Stillman DJ (1997). A large protein complex containing the yeast Sin3p and Rpd3p transcriptional regulators. Mol. Cell Biol..

[44] Rundlett SE (1996). HDA1 and RPD3 are members of distinct yeast histone deacetylase complexes that regulate silencing and transcription. Proc. Natl Acad. Sci. USA.

[45] Wu J, Suka N, Carlson M, Grunstein M (2001). TUP1 utilizes histone H3/H2B-specific HDA1 deacetylase to repress gene activity in yeast. Mol. Cell.

[46] Robyr D (2002). Microarray deacetylation maps determine genome-wide functions for yeast histone deacetylases. Cell.

[47] Carmen AA, Rundlett SE, Grunstein M (1996). HDA1 and HDA3 are components of a yeast histone deacetylase (HDA) complex. J. Biol. Chem..

[48] Lee JH, Maskos K, Huber R (2009). Structural and functional studies of the yeast class II Hda1 histone deacetylase complex. J. Mol. Biol..

[49] Buker SM (2007). Two different Argonaute complexes are required for siRNA generation and heterochromatin assembly in fission yeast. Nat. Struct. Mol. Biol..

[50] Shen H (2016). Structural and histone binding ability characterization of the ARB2 domain of a histone deacetylase Hda1 from *Saccharomyces cerevisiae*. Sci. Rep..

[51] Suraweera A, O’Byrne KJ, Richard DJ (2018). Combination therapy with histone deacetylase inhibitors (HDACi) for the treatment of cancer: achieving the full therapeutic potential of HDACi. Front. Oncol..

[52] Eckschlager Tomas, Plch Johana, Stiborova Marie, Hrabeta Jan (2017). Histone Deacetylase Inhibitors as Anticancer Drugs. International Journal of Molecular Sciences.

[53] Koutsounas I, Giaginis C, Theocharis S (2013). Histone deacetylase inhibitors and pancreatic cancer: are there any promising clinical trials?. World J. Gastroenterol..

[54] Poligone B, Lin J, Chung C (2011). Romidepsin: evidence for its potential use to manage previously treated cutaneous T cell lymphoma. Core Evid..

[55] Day JA, Cohen SM (2013). Investigating the selectivity of metalloenzyme inhibitors. J. Med. Chem..

[56] Guo C, Mi J, Brautigan DL, Larner JM (2007). ATM regulates ionizing radiation-induced disruption of HDAC1:PP1:Rb complexes. Cell Signal.

[57] Verdin E, Dequiedt F, Kasler HG (2003). Class II histone deacetylases: versatile regulators. Trends Genet.

[58] Yang XJ, Gregoire S (2005). Class II histone deacetylases: from sequence to function, regulation, and clinical implication. Mol. Cell Biol..

[59] Fischle W (2001). Human HDAC7 histone deacetylase activity is associated with HDAC3 in vivo. J. Biol. Chem..

[60] Mathias RA, Guise AJ, Cristea IM (2015). Post-translational modifications regulate class IIa histone deacetylase (HDAC) function in health and disease. Mol. Cell Proteom..

[61] Parra MClassIIa (2015). HDACs—new insights into their functions in physiology and pathology. FEBS J..

[62] Park SY (2018). Structural basis of the specific interaction of SMRT corepressor with histone deacetylase 4. Nucleic Acids Res..

[63] Bottomley MJ (2008). Structural and functional analysis of the human HDAC4 catalytic domain reveals a regulatory structural zinc-binding domain. J. Biol. Chem..

[64] Schuetz A (2008). Human HDAC7 harbors a class IIa histone deacetylase-specific zinc binding motif and cryptic deacetylase activity. J. Biol. Chem..

[65] Burli RW (2013). Design, synthesis, and biological evaluation of potent and selective class IIa histone deacetylase (HDAC) inhibitors as a potential therapy for Huntington’s disease. J. Med. Chem..

